# Draft Genome Sequence of a *Mannheimia haemolytica* Serotype 6 Isolate Collected from the Nasopharynx of a Beef Calf with Bovine Respiratory Disease

**DOI:** 10.1128/genomeA.00051-13

**Published:** 2013-04-04

**Authors:** Cassidy L. Klima, Shaun R. Cook, Kristen R. Hahn, Kingsley K. Amoako, Trevor W. Alexander, Steve Hendrick, Tim A. McAllister

**Affiliations:** a Agriculture and Agri-Food Canada Research Centre, Lethbridge, Alberta, Canada; b Department of Large Animal Clinical Science, Western College of Veterinary Medicine, University of Saskatoon, Saskatoon, Saskatchewan, Canada; c Canadian Food Inspection Agency, National Centres for Animal Disease, Lethbridge Laboratory, Lethbridge, Alberta, Canada

## Abstract

The draft genome of a *Mannheimia haemolytica* serotype 6 isolate obtained from the nasopharynx of a feedlot calf with bovine respiratory disease is described.

## GENOME ANNOUNCEMENT

*Mannheimia haemolytica*, a member of the family *Pasteurellaceae*, is a commensal in the upper respiratory tract of birds and mammals and can contribute to acute fibrinous pleuropneumonia. In feeder cattle, this is termed bovine respiratory disease (BRD) and costs the North American industry millions of dollars yearly through treatment, reduced meat yield, and mortalities.

Host specificity and virulence of *M. haemolytica* are linked to serovars with serotypes 1 (S1) and 6 (S6) being infectious to cattle (1). Whole genomes of serotypes 1 (2) and 2 (S2) (3) have been sequenced, but not that of S6. We describe a draft genome sequence of *M. haemolytica* H23, an S6 isolate obtained from the nasopharynx of a beef calf with BRD.

The *M. haemolytica* H23 genome was sequenced by Cofactor Genomics (St. Louis, MO) using both Roche 454 Genome Sequencer (GS) Junior and Illumina Genome Analyzer IIx. A total of 99,677 single-end 400-bp 454 reads and 47,372,771 paired-end 80-bp Illumina reads were generated, with respective coverages of 14× and 2,847×. Independent assemblies using Newbler 2.5p1 (454) and SOAP*denovo* (Illumina) were merged with Minimus2. A draft genome of 69 contigs with a total of 2,662,064 bp, a G+C content of 40.8%, and an N_50_ of 101,714 bp was produced and verified using optical mapping.

Gene prediction and function using the Integrated Microbial Genomes (IMG) (http://img.jgi.doe.gov/) platform (4) identified 2,704 genes representing 89.2% of total base pairs. Of these, 2,628 are protein-coding sequences, and 2,197 have assigned functions, including 416 for transporters, 764 for signal peptides, and 551 for transmembrane proteins. Compared to S1 and S2 genomes, 52 genes coding for primarily hypothetical or phage-related proteins are exclusive to *M. haemolytica* H23. A clustered regularly interspaced short palindromic repeat (CRISPR) element and a type III secretory component identical to S2 (3) were also detected. Gene prediction identified 3 5S rRNA genes, 1 16S rRNA gene, and 1 23S rRNA gene.

### Nucleotide sequence accession numbers.

The draft genome sequence of *M. haemolytica* H23 has been included in the GenBank Whole-Genome Shotgun (WGS) database under the accession no. AOGP00000000. The version described here is the first version, accession no. AOGP01000000.
